# Autoantibody profiles are associated with specific clinical features in psychotic disorders

**DOI:** 10.1192/j.eurpsy.2021.2026

**Published:** 2021-08-13

**Authors:** K.O. Schubert, A. Jernbom Falk, C. Galletly, D. Just, C. Toben, B. Baune, S. Clark, D. Liu, P. Nilsson, A. Manberg

**Affiliations:** 1 Discipline Of Psychiatry, The University of Adelaide, Adelaide, Australia; 2 Community Mental Health, Northern Adelaide Mental Health Services, Salisbury, Australia; 3 Science For Life Laboratory, Royal Institute of Technology, Stockholm, Sweden; 4 The University Of Melbourne, The Florey Institute of Neuroscience and Mental Health, Parkville, Australia; 5 Department Of Psychiatry And Psychotherapy, University of Münster, Münster, Germany

**Keywords:** Autoimmunity, Autoantibodies, psychopathology, psychosis

## Abstract

**Introduction:**

Immune system abnormalities exist across a range of psychiatric disorders. Autoimmunity, characterized by the production of antibodies against the body’s own antigens, is a feature of immune system dysfunction and could play a role in mental disorder pathophysiology. Better understanding of the associations of auto-immunoglobulin G (IgG) repertoires with clinical features of mental illness could yield novel models of psychosis pathophysiology and markers for biological patient stratification.

**Objectives:**

To undertake global screening for auto-IgG expression in a large cohort of people with psychotic disorders; to determine whether associations exist between autoantibody expression and clinical features.

**Methods:**

Cross-sectional quantification of auto-IgGs in blood plasma of 461 people with established psychotic disorder diagnoses. For global screening, pooled samples of phenotypically representative patient groups were exposed to planar protein microarrays containing 42,000 human antigens. For targeted profiling, expression levels of 380 autoantibodies were quantified by suspension bead array (SBA) in each patient’s plasma.

**Results:**

We identified highly individual autoantibody profiles with no evidence for co-expression patterns. We found 6 autoantibodies robustly associated with specific psychopathology: anti-AP3B2, detected in 5% of the cohort of whom 100% had persecutory delusions; anti-TDO2 (5% of the cohort, 100% hallucinations); anti-CRYGN (4%, 86% initial insomnia); anti-APMAP (3%, 86% poor appetite); anti-OLFM1 (2.5%, 100% above median cognitive function); and anti-WHAMMP3 (2%, 90% anhedonia and dysphoria). Examination of the auto-IgG binding site on the TDO2 protein revealed a putative pathophysiological mechanism involving the kynurenine pathway.

**Conclusions:**

We identified 6 frequently occurring autoantibodies that were associated with specific clinical features in people with psychotic disorders.

**Disclosure:**

No significant relationships.

